# A Case Report and Literature Review of Adrenal Myelolipoma

**DOI:** 10.7759/cureus.43240

**Published:** 2023-08-09

**Authors:** Cristobal S Duarte Regalado, José I Guzmán Mejía, Gabriela E Gutiérrez Uvalle, Angie E Vargas Rodríguez, Jeanille González Ledo

**Affiliations:** 1 Surgery, General Hospital of Mexico Dr. Eduardo Liceaga, Mexico City, MEX

**Keywords:** open surgery, abdominal pain, adrenal gland, adrenal myelolipoma, incidentaloma

## Abstract

Adrenal myelolipoma is considered a benign neoplasm that accounts for 6% to 16% of adrenal incidentalomas, and it is the second most common incidental adrenal tumor after adrenal adenomas. They are usually asymptomatic; however, in the presence of symptoms, significant growth, or complications, open surgical resection is indicated. We present the case of a 46-year-old woman with obesity and diabetes who experienced five years of left hemiabdominal pain, which was unsuccessfully treated symptomatically. A computed tomography scan revealed findings suggestive of pancreatic lipoma and a suggestive image of left adrenal myelolipoma. Resection of the tumor was performed using an anterior midline approach, and histopathological examination confirmed left adrenal myelolipoma. The presented case represents the typical presentation of these tumors in a patient in the fifth decade of life with obesity, diabetes, and nonspecific abdominal pain possibly related to the size of the lesion found. Surgical intervention was indicated due to the presence of symptoms, lesion size, contiguity with abdominal organs, and the absence of a precise diagnosis. An anterior midline approach was chosen, and histopathological examination provided a definitive diagnosis. Adrenal myelolipoma is a rare entity that is often asymptomatic and incidentally diagnosed through imaging studies. However, they should be resected when symptomatic to prevent complications. Open surgical resection is the preferred approach.

## Introduction

Adrenal myelolipoma is a benign neoplasm composed of adipose and myeloid tissue [1,2]. It accounts for 6% to 16% of adrenal incidentalomas and is the second most common cause after adrenal adenomas, with an approximate autopsy prevalence of 0.08% to 0.2% [3,4]. It originates from metaplastic changes in mesenchymal cells or as a result of overstimulation by adrenocorticotropic hormone (ACTH) [5,6]. Clinically, myelolipomas can be asymptomatic or present with abdominal pain or nonspecific symptoms such as fever or weight loss [7,8]. Computed tomography and magnetic resonance imaging are the diagnostic imaging modalities of choice [9,10]. Surgical resection is indicated in cases of significant growth, symptomatic presentation, or hormonal hypersecretion, with open surgical approach being the most commonly performed method (88.4% of cases) [11,12].

## Case presentation

**Figure 1:**
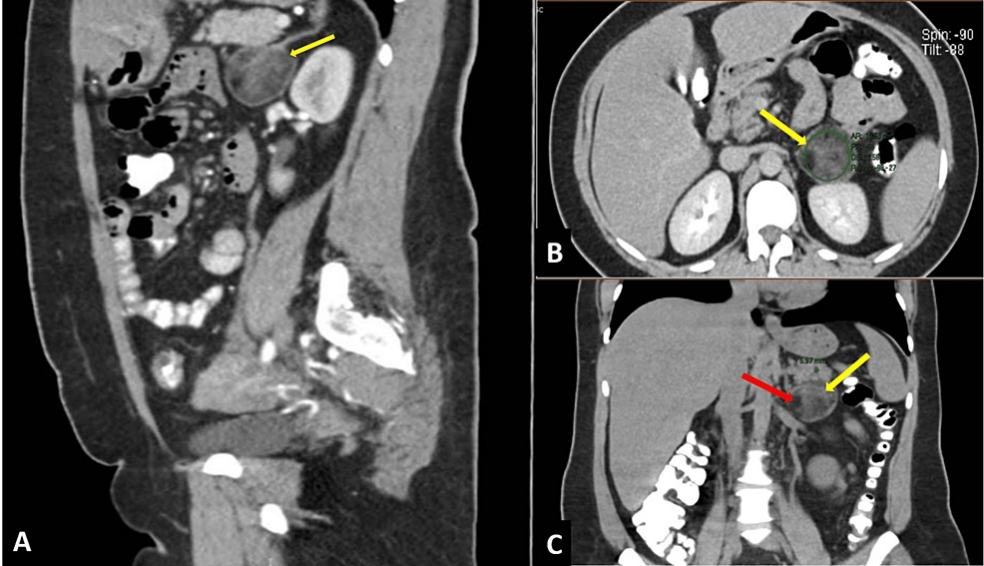
CT Scan of the Abdomen and Pelvis A) Sagittal section: The tumor (yellow arrow) maintains a separation plane with adjacent structures (small intestine, pancreas, left kidney, and adrenal gland). B) Axial section: A solid tumor (yellow arrow) with a thin and well-defined wall is observed, showing a heterogeneous pattern with areas of up to -60 Hounsfield Units (HU). C) Coronal section: The interior of the tumor is heterogeneous, with hypodense areas corresponding to adipose tissue (yellow arrow) and hyperdense areas corresponding to myeloid tissue (red arrow), measuring 4.3x4.6x4.7 cm in dimensions.

**Figure 2:**
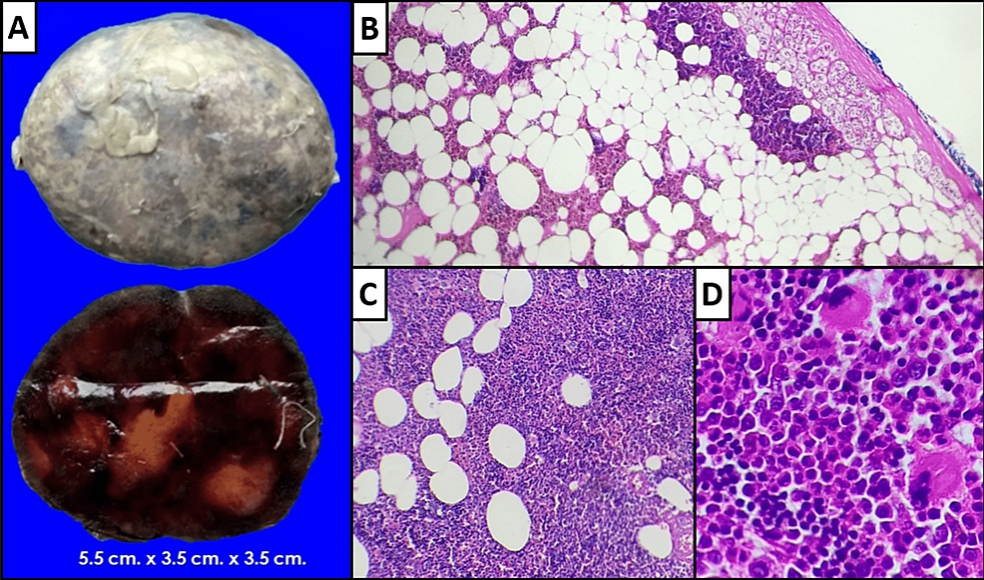
Pathological Anatomy A) Macroscopic photographs of the external surface, corresponding to the capsule and a cut section with a reddish-brown hemorrhagic appearance, with yellow areas resembling adipose tissue. B) Photomicrograph (hematoxylin-eosin, 10x) showing residual adrenal cortex with an underlying neoplasm. C) Photomicrograph (hematoxylin-eosin, 10x) revealing a neoplasm with two components, one of which consists of mature adipocytes mixed with hematopoietic components. D) Photomicrograph (hematoxylin-eosin, 40x) providing a closer view of the hematopoietic elements, with islands of erythropoiesis, myeloid precursors, and megakaryocytes being identified.

We present the case of a 46-year-old woman with a history of type 2 diabetes treated with metformin, laparoscopic cholecystectomy for cholelithiasis, and scheduled cesarean section. The patient started experiencing left flank pain radiating to the left hypochondrium five years ago. The pain was accompanied by abdominal distension. She initially sought care from a colorectal surgeon who performed a colonoscopy, which revealed Kudo I polyps versus subepithelial lesion in the ascending colon. The patient received symptomatic analgesic treatment with partial improvement and was later referred to our service and attended an outpatient consultation. On physical examination, the patient had grade I obesity (weight: 76 kg; height: 158 cm; body mass index: 30.5). The abdomen was distended due to adipose tissue, with a Pfannenstiel scar. Normal bowel sounds were auscultated, and there was no superficial tenderness, but deep palpation elicits pain predominantly in the epigastric and left hypochondriac regions. There was no visceromegaly or signs of peritoneal irritation. A computed tomography scan was recommended to rule out colorectal pathology, which revealed a hypodense image in the pancreatic tail without enhancement after contrast administration, measuring 5.9x5.6x4.0 cm. The left adrenal gland's lateral arm showed loss of typical morphology due to a thin-walled and well-defined solid lesion, internally heterogeneous with poorly defined areas of higher attenuation up to -60 Hounsfield Units (HU), enhancing to -15 HU after contrast administration, measuring 4.3x4.6x4.7 cm (Figure 1). Initial laboratory results were within normal ranges (Table 1). After anesthesia and cardiovascular assessment, the patient was scheduled for tumor resection using an open technique through a suprainfraumbilical midline incision. Intraoperatively, a solid, ovoid-shaped, wine-colored encapsulated tumor measuring 7x5x5 cm (Figure 2) was found in the upper pole of the left kidney and the tail of the pancreas, without evidence of local infiltration. Complete resection was performed with a blood loss of 50 ml and a surgical time of 123 minutes, without complications in the postoperative period. The patient tolerated diet and ambulation after 12 hours and was discharged in stable condition after two days. Follow-up in the outpatient clinic showed an asymptomatic patient engaged in daily activities. The histopathological study confirmed a left adrenal myelolipoma.

**Table 1:** Laboratory Study Results on Admission CRP: C-reactive protein, PT: prothrombin time, INR: international normalized ratio.

Analyte	Patient´s Analyte	Normality Ranges
Leucocytes	9.2	4.5-10 x 10^3^/µL
Hemoglobin	12	12-16 g/dL
Platelets	386	150-450 x 10^3^/µL
Glucose	85	74-106 mg/dL
Creatinine	0.68	0.66-1.09 mg/dL
Total bilirubin	0.39	0.1-1.2 mg/dL
Amylase	48	30-110 U/L
Lipase	35	10-140 U/L
Sodium	135	135-145 mmol/L
Potassium	4.2	3.5-5 mmol/L
Calcium	8.74	8.5-10.3 mg/dL
Phosphorus	3	2.5 4.5 mg/dL
Magnesium	1.9	1.9-2.5 mg/dL
Albumin	4.3	3.5-5.2 g/dL
Cortisol	10	3-10 µg/dL
Insulin	19.27	5-25 µU/mL
CRP	9.64	0-19 mg/dL
PT	12.1	11-13.5 s
INR	0.9	0.8-1.2 s

## Discussion

The first case of this entity was presented by Gierke in 1905, and in 1926, Oberling named it myelolipoma. The case presented corresponds to a patient with a left adrenal myelolipoma, which is a benign tumor characterized by the presence of adipose and hematopoietic tissue. Its prevalence ranges from 0.08% to 0.2% in histopathological studies, and it is the second most common incidental adrenal tumor, accounting for 15% of cases [13,14]. The hypotheses regarding its formation involve stimuli such as necrosis or inflammation that could lead to metaplasia of reticuloendothelial cells. There is a strong association between myelolipoma and conditions such as Cushing's disease, pheochromocytoma, obesity, and diabetes. Additional endocrine evaluation for pheochromocytoma and Cushing's disease was not performed, as most authors suggest not conducting such studies when the lesion has typical myelolipoma characteristics in imaging, as was the case with our patient. Additionally, the patient did not have the financial means to undergo further testing [15]. Myelolipomas typically occur in adulthood, with a median age of 51 years, without gender predilection. They are usually unilateral with a slight preference for the right side, and their size can vary from a few millimeters up to 43 cm [3]. Myelolipomas consist predominantly of fatty areas mixed with hematopoietic tissue, as observed in the photomicrographs of our case. The most common presenting symptoms are abdominal pain (22%), pain in the hypochondrium (13.9%), abdominal mass (5.2%), fever, weight loss, and virilization. Four clinicopathological patterns of adrenal myelolipomas are known: isolated adrenal myelolipoma, adrenal myelolipoma with acute hemorrhage, extra-adrenal myelolipoma, and adrenal myelolipoma with associated adrenal disease [1,16]. Ultrasonography, computed tomography, and magnetic resonance imaging support the diagnosis, with computed tomography being the most sensitive in identifying fat density within the lesions. The differential diagnosis of retroperitoneal tumors containing fat includes benign adrenal adenoma, adrenal carcinoma, retroperitoneal liposarcoma, and renal angiomyolipoma. The management of adrenal myelolipoma should be based on the size of the lesion and the presence of symptoms [17]. Tumors measuring less than 5 cm and being asymptomatic are generally monitored by computed tomography for a period of one to two years. However, if the tumors become symptomatic or exceed 5 cm in size, elective resection is recommended due to the potential complications of spontaneous rupture or retroperitoneal hemorrhage, which can be fatal [18]. The surgical approach can be chosen based on the tumor size and can include laparoscopic or open techniques, such as transabdominal, lumbar, subcostal, or posterior access laparotomy. Laparoscopic approach is considered the gold standard in the surgical treatment of adrenal myelolipomas smaller than 10 cm, well-defined, and without invasion to neighboring structures. The midline approach is indicated for tumors larger than 10 cm or in cases where there are adhesions and infiltration of surrounding structures. In this case, due to the surgeon's experience and preference, an open approach was chosen. Adrenal myelolipoma is usually a disease with an excellent prognosis, so long-term post-surgical follow-up is not required. However, there are published reports recommending an abdominal ultrasound within a three- to six-month interval and an annual clinical examination after surgery for giant tumors (>10 cm) [19]. The case we have presented can be considered typical in the evolution of these tumors, occurring in a woman in her fifth decade of life with obesity, diabetes, and nonspecific diffuse abdominal pain. Surgery was indicated due to the presented symptoms and the size of the lesion found on computed tomography. The patient is currently under surveillance and asymptomatic.

## Conclusions

Adrenal myelolipoma often remains asymptomatic and is discovered either during the investigation of other pathologies or incidentally through radiological studies. Definitive diagnosis is only achieved through histopathological examination of the surgical specimen. Surgical treatment is recommended for tumors larger than 5 cm, symptomatic tumors, those with adhesions or infiltration of surrounding structures, or when changes in their characteristics during follow-up suggest potential major complications such as malignancy or bleeding.
